# Tresca Stress Simulation of Metal-on-Metal Total Hip Arthroplasty during Normal Walking Activity

**DOI:** 10.3390/ma14247554

**Published:** 2021-12-09

**Authors:** Muhammad Imam Ammarullah, Ilham Yustar Afif, Mohamad Izzur Maula, Tri Indah Winarni, Mohammad Tauviqirrahman, Imam Akbar, Hasan Basri, Emile van der Heide, J. Jamari

**Affiliations:** 1 Department of Mechanical Engineering, Faculty of Engineering, Diponegoro University, Tembalang, Semarang 50275, Central Java, Indonesia; iyustar.afif@gmail.com (I.Y.A.); izzurmaula@gmail.com (M.I.M.); mtauviq99@yahoo.com (M.T.); 2 Undip Biomechanics Engineering & Research Center (UBM-ERC), Diponegoro University, Tembalang, Semarang 50275, Central Java, Indonesia; triwinarni@lecturer.undip.ac.id; 3 Department of Anatomy, Faculty of Medicine, Diponegoro University, Tembalang, Semarang 50275, Central Java, Indonesia; 4 Center for Biomedical Research (CEBIOR), Faculty of Medicine, Diponegoro University, Tembalang, Semarang 50275, Central Java, Indonesia; 5 Department of Mechanical Engineering, Faculty of Engineering, Sriwijaya University, Indralaya 30662, South Sumatra, Indonesia; imamakbarrr0502@gmail.com (I.A.); hasan_basri@unsri.ac.id (H.B.); 6 Laboratory for Surface Technology and Tribology, Faculty of Engineering Technology, University of Twente, Postbox 217, 7500 AE Enschede, The Netherlands; e.vanderheide@utwente.nl

**Keywords:** Tresca stress, metal-on-metal, total hip arthroplasty, normal walking activity

## Abstract

The selection of biomaterials for bearing in total hip arthroplasty is very important to avoid various risks of primary postoperative failure for patients. The current investigation attempts to analyze the Tresca stress of metal-on-metal bearings with three different materials, namely, cobalt chromium molybdenum (CoCrMo), stainless steel 316L (SS 316L), and titanium alloy (Ti6Al4V). We used computational simulations using a 2D axisymmetric finite element model to predict Tresca stresses under physiological conditions of the human hip joint during normal walking. The simulation results show that Ti6Al4V-on-Ti6Al4V has the best performance to reduce Tresca stress by 45.76% and 39.15%, respectively, compared to CoCrMo-on-CoCrMo and SS 316L-on-SS 316L.

## 1. Introduction

Metal-on-metal total hip arthroplasty has been increasingly selected in hip replacement surgery for diseased hips, especially for younger patients with higher activity levels [1]. This is due to the high number of failure cases that have been found in the use of metal-on-polyethylene and ceramic-on-polyethylene bearings, where polyethylene wear induces osteolysis and aseptic loosening. Meanwhile, the use of ceramic-on-ceramic is prone to failure due to cracking due to high-intensity activities, generally carried out by younger users.

Looking at the data published by the Australian Orthopedic Association (AOA) in 2020 [2] shows that the total failure cases of hip arthroplasty with metal-on-metal bearings are relatively higher than other options. Even so, metal-on-metal is still widely used in several developing countries, including Indonesia to meet the domestic market’s need for hip joint implants independently without having to import [3,4,5]. This is based on the advantages of metal-on-metal in terms of relatively affordable prices, easily available raw materials, and limited production equipment.

The main problem with using metal-on-metal is the production of metal ions due to metal wear particles that can spread throughout the body from the bloodstream. These metal ions cause poisoning and various negative reactions in the human body system. Efforts that can be made to prevent implants from failing are the further evaluation of the choice of metal material used for metal-on-metal total hip arthroplasty. Several metal materials are commonly used for metal-on-metal bearings, including cobalt chromium molybdenum (CoCrMo), 316 L stainless steel (SS 316L), and titanium alloy (Ti6Al4V) [6].

In evaluating the performance of total hip arthroplasty, several previous studies used the von Mises stress, such as that conducted by Chethan et al. [7] and Carreiras et al. [8]. However, the use of Tresca stress is considered to be better than the von Mises stress because the Tresca failure theory has a smaller safety area than the von Mises failure theory, so it can be said that the use of Tresca is safer than of von Mises [9]. Research related to the evaluation of the performance of artificial joints with Tresca stress has previously been carried out by Usman and Huang [9] and Abdullah et al. [10]. Unfortunately, the evaluation of the use of materials for metal-on-metal total hip arthroplasty with Tresca stresses has not yet been carried out.

To accommodate this problem, the current study focuses on evaluating the Tresca stress in metal-on-metal bearings with different materials. A finite element-based prediction model has been created to solve problems using computational simulations. Gait loading has been used to reflect Tresca stress conditions more accurately in daily activities for simulating metal-on-metal total hip arthroplasty.

## 2. Materials and Methods

### 2.1. Geometric Parameters and Material Properties

The bearing geometry used for the current study was adopted from the research conducted by Jamari et al. [3] and is described in Table 1 for both the femoral head and the acetabular cup, which are commonly used in total hip arthroplasty.

The properties of metallic materials evaluated in the current study were based on previous studies: CoCrMo from Jamari et al. [3] and SS 316L and Ti6Al4V from Jiang et al. [11], which are described in Table 2. All simulated materials are assumed to be homogeneous, isotropic, and linear elastic.

To consider the surface roughness during articulation in metal-on-metal bearings, the coefficient of friction is given for the current computational simulations described in Table 3. The coefficient values for various metal-on-metal bearings were adopted from the previous literature: CoCrMo-on-CoCrMo from Jamari et al. [3], SS 316L-on-SS 316L from Jin et al. [12], and Ti6Al4V-on-Ti6Al4V from Arash et al. [13].

### 2.2. Finite Element Modelling

**Figure 1:**
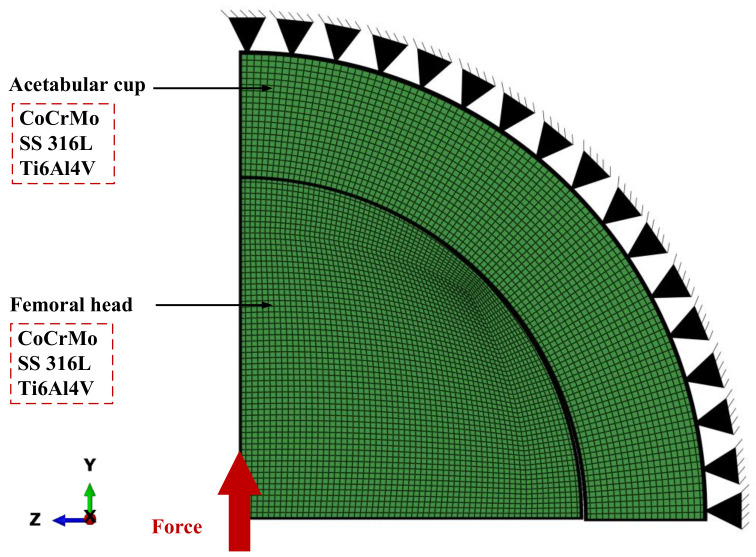
Metal-on-metal total hip arthroplasty model for finite element analysis.

A computational model is established to analyze metal-on-metal bearings is carried out by analyzing two main components, namely, the acetabular cup and femoral head by adopting a 2D axisymmetric ball-in-socket model as shown in Figure 1. Pelvic bones, fixation system, and femoral stem components were not considered in the current study to simplify the computational process but without significantly affecting the results. The definition of contact was made by configuring the femoral head and acetabular cup contact surfaces as master and slave surfaces, respectively. For contact formulation, discretization method, friction formulation, and threshold are defined as surface to surface, penalty, and nodal, respectively.

The contact process also ignores micro separation with the femoral head always concentric with the acetabular cup position. The influence of synovial fluid during contact was ignored in the analysis with only considering surface roughness in dry contact. Temperature changes are not analyzed. Then, the acetabular cup component is made immobile by selecting fix constraint set of nodes in the outer acetabular cup area. In addition, the loading condition is given, being the concentrated load on the femoral head with selecting center node of femoral head.

The selection of the number of elements is obtained through a convergence study to find the optimal number of elements using the H-refinement method by finding the least number of elements but with near-accurate results to provide computational time efficiency. A total of 5500 CAX4 elements have been used with details of 2000 and 3500 CAX4 elements for the femoral head and acetabular, respectively, in analyzing Tresca stress on metal-on-metal using ABAQUS/CAE 6.14-1 software. In the present computational simulation, Tresca stress is analyzed in the bulk area of the acetabular cup.

### 2.3. Gait Cycle

**Figure 2:**
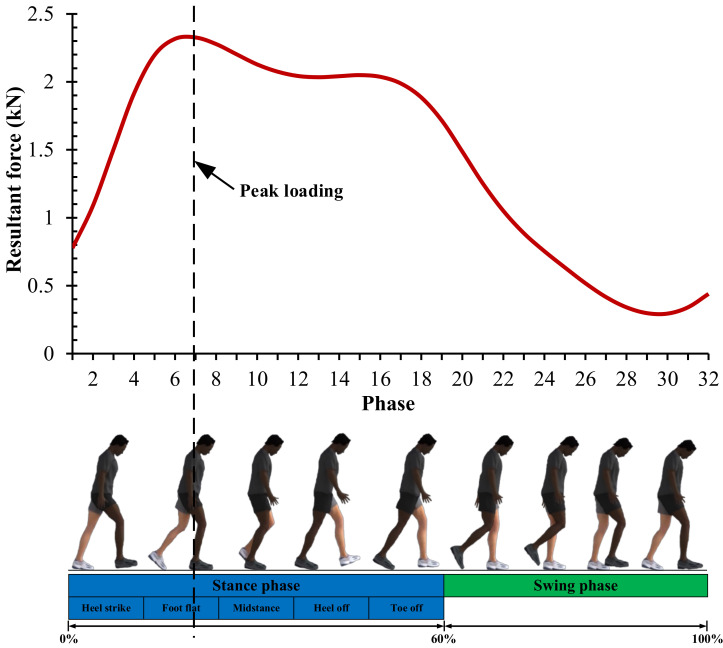
Gair loading based on normal walking condition [3].

Computational simulation is carried out with physiological loading by adopting normal walking conditions that is the most common activity carried out by patients who have performed total hip replacement surgery. However, in our present computational simulation, we only consider gait loading in the form of resultant force and ignore the range of motion effect for model simplification as conducted by Basri et al. [14]. This was done to focus the present study on the effect of resultant force under normal walking conditions against Tresca stress. The normal walking condition with one full cycle simplified into 32 phases has been adopted from the previous study by Jamari et al. [3] shown in Figure 2. In one loading cycle consisting of the ‘stance phase’ which is the first 19 phases and continued with the ‘swing phase’ to completion, the resultant force is highest in the seventh phase of 2326 N that has 2.5–3 times the average human body weight in general. Then, the lowest phase occurs in the 30th phase.

## 3. Results and Discussion

**Figure 3:**
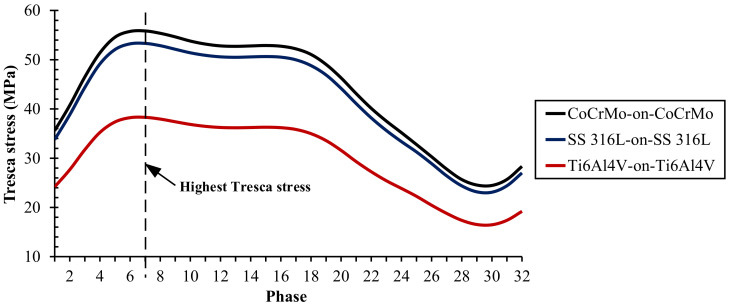
Maximum Tresca stress for different metal-on-metal bearing materials in full cycle.

**Figure 4:**
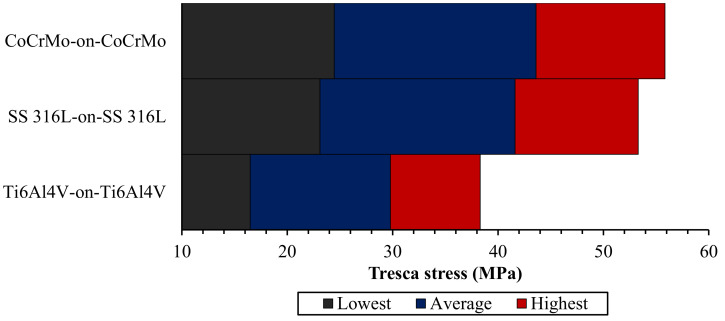
Comparison of highest, average, and lowest Tresca stress of metal-on-metal bearings materials on under normal walking condition.

The maximum Tresca stress from metal-on-metal bearing with different metallic materials during normal walking cycle is presented in Figure 3. The comparison of the highest, average, and lowest Tresca stresses of metal-on-metal bearing materials under normal walking conditions are described in Figure 4. The maximum Tresca stress value changes due to the difference in the resultant force applied during loading in the normal walking condition, where the highest maximum Tresca stress value is in the seventh phase for all metal-on-metal bearings in this study.

Ti6Al4V-on-Ti6Al4V has the lowest Tresca stress value of 38.31 MPa. Tresca stress values were found to increase in CoCrMo-on-CoCrMo and SS 316L-on-SS 316L bearings by about 45.76% and 39.15%, respectively, compared to Ti6Al4V-on-Ti6Al4V. Apart from the magnitude of resultant force applied, Young’s modulus also plays a significant role in the Tresca stress results. The greater Young’s modulus of material under the same loading conditions will give a higher Tresca stress. Therefore, metal-on-metal bearings with CoCrMo material have the highest Tresca stress value under normal walking conditions compared to other bearing materials and vice versa for metal-on-metal bearings using Ti6Al4V material. The maximum Tresca stress values of various metal-on-metal bearings can be seen in Table 4.

**Figure 5:**
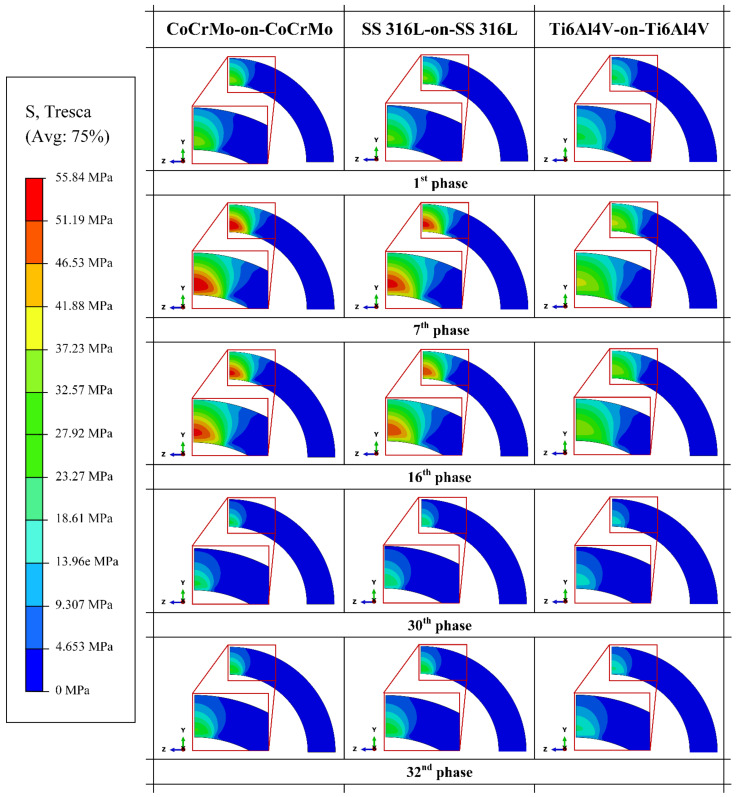
Tresca stress distribution on the acetabular cup for different metal-on-metal bearing materials at selected phases.

Figure 5 illustrates the distribution contour of Tresca stress from Tresca terminology in ABAQUS [15]. The Tresca stress distribution contour is displayed using 5 selected phases from 32 phases in a normal walking cycle, namely the 1st phase that is the initial cycle, the 7th phase that is the phase with the highest gait loading, the 16th phase that is the middle of the cycle, the 30th phase that is the phase with the lowest gait loading, and the 32nd phase that is the end of the cycle. It can be seen that the distribution contour of the Tresca stress will be wider and the value of the Tresca stress will be higher along with the greater the resultant force applied.

**Figure 6:**
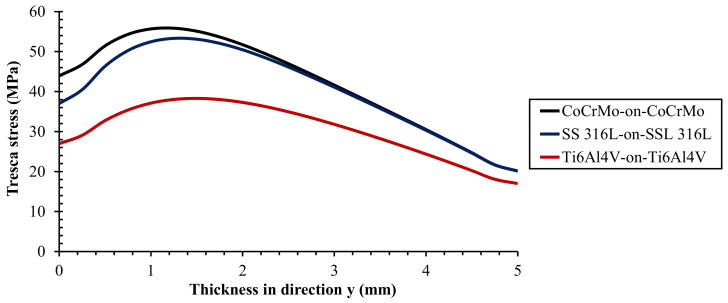
Tresca stress profile as a function of acetabular cup thickness for different metal-on-metal bearing materials at peak loading.

**Figure 7:**
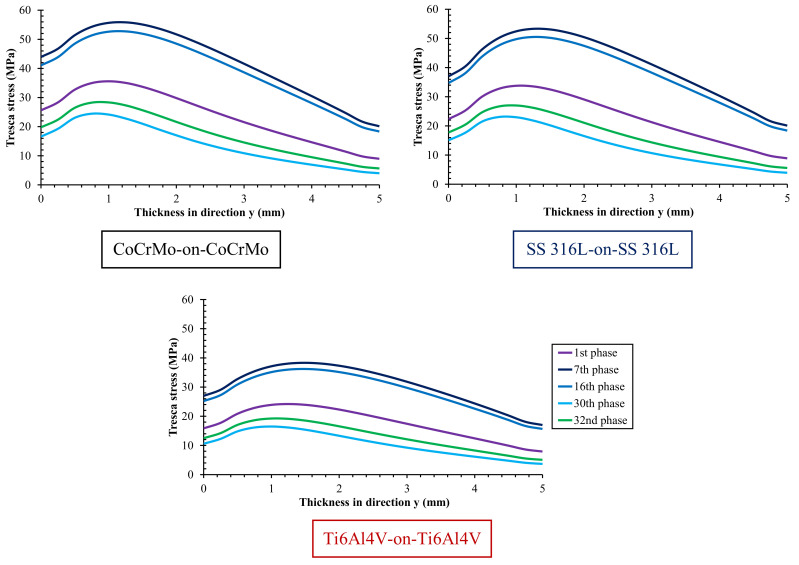
Tresca stress profile as a function of acetabular cup thickness for different metal-on-metal bearing materials at selected phases.

The relationship between Tresca stress and acetabular cup thickness on metal-on-metal bearings with different metallic materials during the peak phase and selected phase is illustrated in Figure 6 and Figure 7. The highest Tresca stress does not occur in the surface contact but in the bulk area that is very prone to losing its elastic properties. In addition, the higher Tresca stress experienced by a material, the higher the probability of failure based on the Tresca failure theory [16]. In the results obtained, it is found that Ti6Al4V-on-Ti6Al4V has the lowest probability of failure with the lowest Tresca stress compared to the other two metal-on-metal bearings.

From the Tresca stress results obtained in the current implant design, various development efforts and further studies for metal-on-metal bearings need to be carried out to reduce the probability of failure. Apart from the selection of materials that have been carried out in the current study, several other efforts can be made, namely, examining the geometry parameters [14,17], the application of textured surfaces [18], and the use of coating layers [19]. The technical aspects of the surgeon’s total hip replacement surgery with total hip arthroplasty also contributes to the long-term survival of the implant [20].

There are several focuses of attention to be conveyed regarding the limitations in our study. First, the coefficient of friction used is constant to represent the effect of lubrication that occurs, where the coefficient of friction should have a value that varies with time [13]. Second, the finite element model used is simple with a two-dimensional model that can reduce the accrual of the results compared with the results from three-dimensional model that are closer to the actual conditions [3]. Finally, the computational simulation does not take into account the range of motion during a normal walking cycle by only analyzing vertical loads that are irrelevant to actual conditions [11].

## 4. Conclusions

The evaluation of Tresca stress on metal-on-metal bearings with different metal materials using a 2D axisymmetric finite element prediction model has been successfully presented in our paper. The highest maximum Tresca stress is obtained in the seventh phase, where the resultant force is highest when conditions are normal walking. The distribution of the Tresca stress contour also widens along with the higher Tresca stress values experienced. The Tresca stress values also correlate with failure probability based on the Tresca failure theory, where we found Ti6Al4V-on-Ti6Al4V bearings to be the most superior among other metal-on-metal bearing options for reducing Tresca stresses.

## Figures and Tables

**Table 1:** Geometric parameters for bearing components.

Parameter	Size (mm)
Femoral head diameter	28
Radial clearance	0.05
Acetabular cup thickness	5

**Table 2:** Material properties for metallic materials.

Material	Young’s Modulus (GPA)	Poisson’s Ratio (-)
CoCrMo	210	0.3
SS 316L	193	0.3
Ti6Al4V	110	0.3

**Table 3:** Coefficient of friction for different materials of metal-on-metal.

Bearings	Coefficient of Friction (-)
CoCrMo-on-CoCrMo	0.2
SS 316L-on-SS 316L	0.8
Ti6Al4V-on-Ti6Al4V	1

**Table 4:** Maximum Tresca stress for different metal-on-metal bearing materials at peak loading.

Bearing Material	Tresca Stress (MPa)
CoCrMo-on-CoCrMo	55.84
SS 316L-on-SS 316L	53.31
Ti6Al4V-on-Ti6Al4V	38.31

## Data Availability

The data presented in this study are available on request from the corresponding author.
